# Immunomodulatory Effects of Multi‐Strain Probiotic Capsules for Psoriatic Arthritis: A Pilot Double‐Blind Randomized Controlled Trial

**DOI:** 10.1002/fsn3.71132

**Published:** 2025-11-05

**Authors:** Ahmed Hussein Hasan Alshihmani, Hanieh Kolahdooz, Mahmoud Mahmoudi, Zahra Rezaieyazdi, Ramiar Kamal Kheder, Nafiseh sadat Tabasi, Afsane Fadaee, Seyed‐Alireza Esmaeili

**Affiliations:** ^1^ Immunology Research Center Mashhad University of Medical Sciences Mashhad Iran; ^2^ Immunology Department, Faculty of Medicine Mashhad University of Medical Sciences Mashhad Iran; ^3^ Rheumatic Diseases Research Center Mashhad University of Medical Sciences Mashhad Iran; ^4^ Medical Laboratory Science Department, College of Science University of Raparin Rania Iraq; ^5^ Department of Medical Analysis, Faculty of Applied Science Tishk International University Erbil Iraq

**Keywords:** B cell, IFNγ, IL‐10, IL‐4, probiotics, psoriasis arthritis, TGFβ, Th1

## Abstract

Psoriatic arthritis (PsA) is characterized by joint inflammation and is frequently associated with psoriasis. Gut dysbiosis has been implicated in PsA pathogenesis, raising interest in probiotics as potential immunomodulatory agents. In a pilot, double‐blind, randomized, placebo‐controlled trial, 14 adults aged 18–60 years with mild‐to‐moderate psoriatic arthritis (PsA; Disease Activity in Psoriatic Arthritis [DAPSA] < 28) were randomized to receive either probiotic capsules or placebo daily for 12 weeks. The probiotic group received a multi‐strain cocktail with a total concentration of 1 × 10^9^ CFU (including *Lactobacillus rhamnosus, Lactobacillus plantarum, Lactobacillus casei, Lactobacillus helveticus, Lactobacillus acidophilus, Bifidobacterium bifidum, Lactobacillus bulgaricus, Lactobacillus gasseri, Bifidobacterium lactis, Bifidobacterium longum, and Streptococcus thermophilus*). The placebo group received lactose‐based inert capsules for the same period. Immune cell populations (CD4^+^ IFN‐γ T cells, B cells, Th2 cells) and cytokine levels (IFN‐γ, IL‐10, TGF‐β, IL‐4) were assessed. Compared with the placebo, probiotic supplementation resulted in a significant reduction in CD4^+^ IFN‐γ T cells (6% ± 0.82 vs. 3.6% ± 0.8; *p* < 0.001), B cells (14.6% ± 1.05 vs. 8.9% ± 1.7; *p* < 0.0001), and IFN‐γ concentrations (37.5 ± 2.4 pg/mL vs. 29.3 ± 2.6 pg/mL, *p* = 0.016). In addition, a significant increase was observed in IL‐10 (9.4 ± 2.8 pg/mL vs. 99.89 ± 28.1 pg/mL, *p* = 0.0032), TGF‐β (18.1 ± 2.7 pg/mL vs. 30.48 ± 7.7 pg/mL, *p* = 0.0073), and IL‐4 (17.6 ± 6.7 pg/mL vs. 58.3 ± 29.2 pg/mL, *p* = 0.0117). Changes in Th2 cell levels were not statistically significant (*p* = 0.54). A multi‐strain probiotic demonstrated promising immunomodulatory effects in PsA by reducing pro‐inflammatory markers and enhancing regulatory cytokines that can be used as a complementary or alternative treatment for PsA patients.

**Trial Registration:** IRCT20221213056802N1

## Graphical abstract

Psoriatic arthritis (PsA) is a systemic autoimmune disease linked to joint inflammation and skin psoriasis. Gut dysbiosis may contribute to PsA, making probiotic supplementation a potential therapy. In our pilot study, PsA patients received a probiotic cocktail or placebo for 12 weeks. The probiotic group showed reduced inflammatory markers and increased anti‐inflammatory cytokines, suggesting probiotics may offer a complementary natural approach to managing PsA.

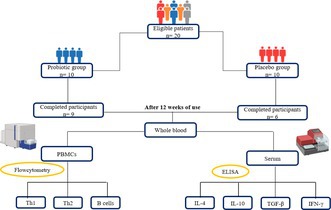

## 1 Introduction

Psoriatic arthritis (PsA) is a systemic autoimmune disease with a complex origin, leading to inflammation and joint deformities in the fingers, spine, and other joints. The global prevalence of psoriatic arthritis (PsA) demonstrates substantial variability, with recent estimates indicating an overall rate of 133 cases per 100,000 individuals (0.13%) in the general population. However, this figure is marked by significant heterogeneity, largely attributed to differences in case definitions, diagnostic criteria, and regional factors (Scotti et al. 2018). Prevalence varies widely across regions: in Japan, it is about 1 per 100,000, while Norway reports 670 per 100,000 (Hoff et al. 2015; Hukuda et al. 2001). PsA is a complex disease resulting from genetic and environmental factors. Studies suggest that 20%–30% of PsA cases may be inherited, as the disease shares genetic similarities with PS through genes in human leukocyte antigens (HLAs) (Prey et al. 2010; Zalesak et al. 2024). An essential aspect of the relationship between PS and PsA lies in the potential predictive factors for PsA development. Research indicates that around 60% of PsA patients initially experienced PS before the onset of joint disease, while the remaining patients experienced both skin and joint manifestations simultaneously (Karmacharya et al. 2022). Environmental factors such as mechanical and metabolic stress, like trauma and obesity, and infections are known to exacerbate PS symptoms (Abdollahi et al. 2021, 2024; Eder et al. 2011). These factors activate immune cells like macrophages and dendritic cells (DC), which present antigens to T lymphocytes (T cells) through Toll‐like receptors (TLRs) (Marzaioli et al. 2022). This interaction releases pro‐inflammatory cytokines, stimulating and differentiating T cells that produce specific disease‐causing molecules. This complex cascade of events ultimately leads to the diverse clinical manifestations observed in PsA patients. Although the immune mechanisms contributing to this disease with impaired immune responses remain unclear, studies have underscored the crucial involvement of immune cells, especially B and T cells (Karczewski et al. 2016; Mavropoulos et al. 2017).

Altered frequencies of peripheral CD4+ T cells, particularly Th1 and Th2 cells, have been observed in patients with PS and PsA compared to healthy individuals. Notably, higher Th1/Th2 ratios have been positively correlated with disease activity in PsA, indicating a more dominant Th1 response during inflammation (Gao et al. 2023). PS patients exhibit significantly higher levels of the Th1 cytokines IFN‐γ and IL‐2 compared to healthy individuals. These elevated Th1 cytokine levels correlate positively with disease severity, suggesting a potential role in the development and progression of psoriasis. PS patients, irrespective of disease activity, display distinct serum cytokine profiles compared to healthy controls, characterized by increased Th1 cytokines (IL‐2, IFN‐γ) and reduced levels of the Th2 cytokine IL‐4 (Jadali et al. 2007; Wang et al. 2022).

While B cell involvement in PsA is less clear than in rheumatoid arthritis (RA), emerging evidence suggests B cells play a pivotal role in the pathogenesis of PsA. These cells function as efficient antigen‐presenting cells, activate T cells, and produce pro‐inflammatory cytokines (Panayi 2005). In RA, B cells are the primary source of rheumatoid factors and other autoantibodies, leading to immune complex formation and complement activation in the joints (Dörner and Burmester 2003). Similarly, in psoriasis, B cells within germinal centers may be stimulated by autoantigens, resulting in autoantibody production and dysregulation of keratinocytes (Noor et al. 2024). Regulatory B cells, capable of inhibiting pro‐inflammatory T cells, may have a crucial role in autoimmune rheumatic diseases. B cells releasing IL‐10 and TGF‐β are reduced during active disease and could serve as biomarkers and possible therapeutic targets in autoimmune rheumatic diseases (Hayashi et al. 2016; Mavropoulos et al. 2017).

Nutritional strategies have been explored as adjunctive approaches in managing psoriasis, with certain dietary patterns demonstrating potential benefits. For instance, low‐calorie diets may be advantageous for individuals with obesity, and gluten‐free regimens are relevant for patients with concurrent celiac disease (Chung et al. 2022). Additionally, micronutrients and natural compounds, such as vitamin D, omega‐3 fatty acids, antioxidants, and curcumin, may exert beneficial effects through modulation of the gut microbiota or by reducing systemic inflammation (Lucius 2022). Emerging evidence underscores the significant involvement of the gut microbiome in the development of psoriatic arthritis (PsA), with disease‐specific alterations in microbial composition increasingly recognized as a contributing factor (Bonomo et al. 2025). Probiotics are known to influence immune responses through both direct and indirect pathways, such as reinforcing the intestinal epithelial barrier, modifying mucus production, and inhibiting colonization by pathogenic microorganisms through competitive exclusion (Mavropoulos et al. 2017). These beneficial microbes also engage in crosstalk with host immune cells and the existing gut flora, playing a key role in maintaining immune equilibrium and modulating specific immune activities (Mazziotta et al. 2023). Although current clinical data on the efficacy of probiotics in rheumatic conditions like PsA remain limited, their immunomodulatory potential offers a possible complement to therapies (Bedaiwi and Inman 2014). In addition, the conventional therapies, such as methotrexate and biological agents like adalimumab and infliximab (anti‐TNFα), are commonly prescribed for mild‐to‐moderate PsA and are often associated with various adverse effects and fail to produce satisfactory clinical outcomes (Armstrong et al. 2013; Pipitone et al. 2003). Methotrexate, for example, can cause gastrointestinal issues and liver toxicity (Ezhilarasan 2021). Moreover, biological agents targeting TNFα may lead to increased susceptibility to infections and other complications (Minozzi et al. 2016). Consequently, alternative therapeutic approaches are being investigated and prescribed. Given the limitations of existing therapies for PsA, there is growing interest in exploring the potential of probiotic supplements as a complementary or alternative treatment option (Lai et al. 2023). Probiotics are living microorganisms that offer health advantages to the host when consumed in appropriate doses. They have been shown to modulate the immune system and reduce inflammation, which may be particularly relevant for individuals with psoriasis (Buhaș et al. 2023). Supplementation with *Lactobacillus rhamnosus* SP1 has been shown to normalize skin gene expression related to insulin signaling and improve adult acne (Fabbrocini et al. 2016), while *Lactobacillus casei* and *Lactobacillus acidophilus* have been reported to significantly improve clinical outcomes in rheumatoid arthritis (Paul et al. 2021), together highlighting the systemic biological effects of probiotics and their potential role in inflammatory disorders. Similarly, in a 12‐week randomized trial of 45 participants with mild‐to‐moderate acne, the addition of probiotics to standard therapy enhanced clinical outcomes and was well tolerated (Jung et al. 2013). Furthermore, a randomized, double‐blind clinical trial in patients with plaque psoriasis demonstrated that a multi‐strain probiotic supplement, including *Lactobacillus acidophilus* , *Lactobacillus casei* , and *Bifidobacterium bifidum* , significantly improved disease severity scores and quality of life indicators compared with placebo (Moludi et al. 2021). Together, these studies highlight the systemic immunomodulatory effects of probiotics and support their potential role as adjunctive therapy in inflammatory disorders.

In light of the potential beneficial effects of probiotics on orchestrating immune response, our research focused on assessing the impact of probiotic supplementation on B‐cell and T‐cell subsets, which are critical players in PsA. Additionally, we examined the influence of probiotics on cytokine production, given their essential role in regulating inflammation and immune cell activation. By elucidating these mechanistic aspects, our findings may contribute to the development of targeted therapeutic strategies incorporating probiotics for the management of psoriatic arthritis and other autoimmune conditions.

## 2 Material and Methods

### 2.1 Eligibility Criteria

This pilot study included adults aged 18–60 years with mild‐to‐moderate psoriatic arthritis (PsA), defined as a Disease Activity in Psoriatic Arthritis (DAPSA) score < 28 (Schoels et al. 2016). Diagnosis was confirmed by an expert rheumatologist at Ghaem Hospital, Mashhad, Khorasan Razavi, Iran. Exclusion criteria were: presence of other inflammatory or autoimmune diseases besides psoriasis and PsA, use of probiotic supplements within 1 month prior to screening, antibiotic treatment within 6 weeks before enrollment, pregnancy or breastfeeding, and planning to become pregnant during the study.

### 2.2 Study Protocol

**FIGURE 1:**
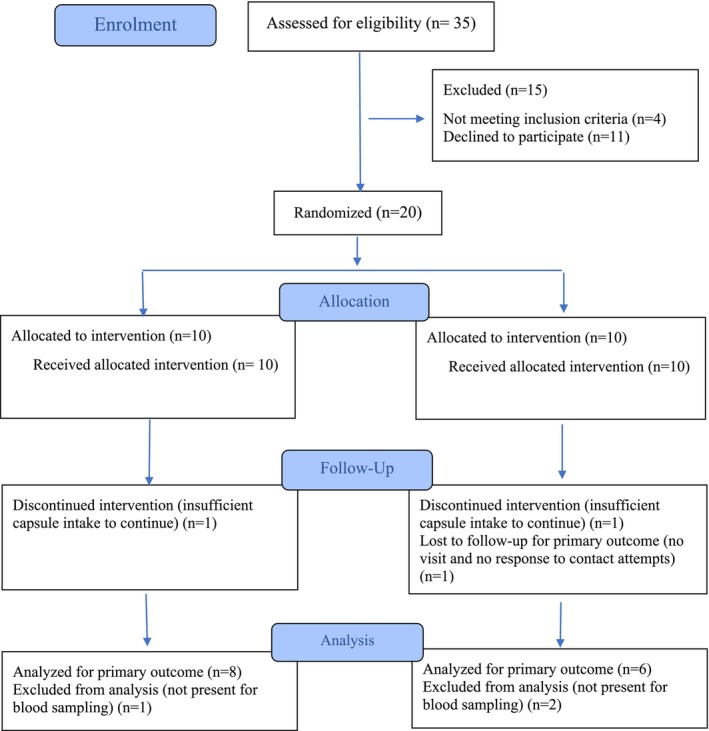
Flow diagram of participants through the pilot clinical trial. Of 35 individuals assessed for eligibility, 15 were excluded. Twenty participants were randomized (probiotic *n* = 10; placebo *n* = 10), all of whom initiated treatment. At follow‐up, 9 in the probiotic group and 8 in the placebo group remained for the primary outcome assessment. Final immunological analyses were performed in 8 probiotic and 6 placebo participants, with attrition due to missed blood sampling and insufficient capsule intake.

This 12‐week, randomized, double‐blind, placebo‐controlled clinical trial was conducted at Ghaem Hospital, Mashhad, Khorasan Razavi, Iran, and initially enrolled 20 patients with PsA, of whom 14 completed the study and were included in the analysis (Figure 1). Participants were randomly assigned to receive either probiotic capsules (*n* = 9) or lactose‐based placebo capsules (*n* = 5) daily for 12 weeks. Probiotic capsules were distributed monthly to ensure freshness and potency, with patients instructed to store them in a refrigerator at home. Manufacturing dates were scheduled to align with monthly distribution to maintain product stability and efficacy. Peripheral blood samples were collected after 12 weeks to assess immunological outcomes. All participants provided written informed consent, and the trial was registered with the Iranian Registry of Clinical Trials (IRCT20221213056802N1) in March 2023. Recruitment occurred between September 1, 2023, and January 15, 2025, with the last follow‐up completed on May 10, 2025.

### 2.3 Randomization and Blinding Procedures

Randomization was performed using a computer‐generated random allocation sequence prepared by an independent researcher not involved in participant recruitment, clinical evaluation, or outcome assessment. Patients were randomly assigned in a 1:1 ratio to receive either probiotic capsules or identical placebo capsules. To ensure blinding, both the probiotics and placebo capsules were manufactured identically in appearance, packaging, and labeling in the pharmaceutical company (Zist Takhmir, Iran). The allocation sequence was concealed in sequentially numbered, opaque, sealed envelopes. The envelopes were opened only after the participant's eligibility had been confirmed and consent obtained. Both participants and investigators, including the rheumatologist performing assessments and laboratory personnel analyzing samples, were blinded to group assignments throughout the study. The blinding code was broken only after the completion of data analysis by an assistant in the pharmaceutical company, unless an emergency required unblinding.

### 2.4 Probiotic and Placebo Formulation

The probiotic capsules contained a proprietary blend of various probiotic strains manufactured by Zist Takhmir Company (LactoCare, Iran). Our multi‐strain probiotic blend (LOT number: PLCO205) included *Lactobacillus rhamnosus* , *Lactobacillus plantarum* , *Lactobacillus casei* , *Lactobacillus helveticus* , *Lactobacillus acidophilus* , *Bifidobacterium bifidum* , *Lactobacillus bulgaricus* , *Lactobacillus gasseri* , *Bifidobacterium lactis* , *Bifidobacterium longum* , and *Streptococcus thermophilus* , with a total concentration of 10^9^ CFU that is evenly distributed across strains according to the WHO guideline (Veterans Affairs (.gov) n.d.). Fructooligosaccharides (FOS) were added at a concentration of 21 mg as a prebiotic. The placebo capsules (LOT number: PLCO204) consisted of inert ingredients: lactose monohydrate, talc, magnesium stearate, maltodextrin, Aerosil, microcrystalline cellulose, and sodium starch glycolate. Lactose monohydrate was chosen as an inert filler to match the probiotic capsule's appearance and texture; the quantity used was minimal and not expected to impact gut microbiota. The Certificate of Analysis (COA) for probiotic (Lot PLCO205) is provided in the Supporting Information section and summarizes finished product specifications, acceptance criteria, observed results, and references used during testing.

### 2.5 Sample Size

This study has been designed as a pilot trial to explore the potential effects of multi‐strain probiotic supplementation on clinical symptoms and inflammatory markers in patients with psoriatic arthritis. Given the exploratory nature and limited availability of eligible patients during the study period, a convenience sample of 14 patients was enrolled. While no formal power calculation was performed, this sample size was considered sufficient to assess feasibility, safety, and generate preliminary efficacy data for future larger‐scale studies.

### 2.6 Flow Cytometry Analysis

Peripheral blood mononuclear cells (PBMCs) were isolated from whole blood using density gradient centrifugation (Ficoll‐Paque Plus, Cedarlane Labs), as described (Esmaeili et al. 2018). Surface staining was performed using anti‐CD4‐FITC (clone SK3, BioLegend) and anti‐CD19‐PE (clone HD37, IQ Products). Intracellular staining was performed using the eBioscience Intracellular Staining Kit, following the manufacturer's instructions, with anti‐IL‐4‐PE (clone MP4‐25D2, BioLegend) and anti‐IFN‐γ‐PE (clone W19227A, BioLegend). Appropriate isotype controls were included to ensure staining specificity. Data were analyzed using FlowJo software. Gating was based on FSC/SSC, followed by CD4+ or CD19+ populations. IL‐4 and IFN‐γ expression was assessed within these gates.

### 2.7 ELISA Analysis

Following the treatment period, serum samples were gathered from all patients and kept at −80°C until they were analyzed. The cytokines of interest, including IFNγ, IL‐4, IL‐10, and TGFβ concentrations, were determined using ELISA kits (Karmania Pars Gene, Iran). All assays were executed following the manufacturer's manual, and samples were measured using a microplate reader (EQ04 Emperor, China) at 450 nm. Standard curves and calculations utilized 4‐parameter logistic regression (4PL), duplicating all measurements.

### 2.8 Statistical Analysis

Data are presented as mean ± SD for normally distributed variables or median (interquartile range) for skewed data. Normality was assessed using the Shapiro–Wilk test and Q–Q plots. Between‐group comparisons (probiotic vs. placebo) were performed using the independent‐samples t‐test for normally distributed data or the nonparametric Mann–Whitney U test for non‐normal data. Given the small sample size, nonparametric methods were prioritized where appropriate. To address multiple comparisons, the Benjamini–Hochberg procedure was applied to control the false discovery rate. Bonferroni correction was used in confirmatory comparisons with limited outcomes. Missing data were handled using complete‐case analysis. All statistical analyses were performed using GraphPad Prism version 10 (GraphPad Software, San Diego, CA, USA), with significance set at *p* < 0.05 unless adjusted for multiple testing.

#### 2.8.1 Harms

Harms were assessed systematically at each visit and via structured interviews, including gastrointestinal discomfort, allergic reactions, or any other adverse events. No adverse events or side effects were reported during the trial or follow‐up, and all participants tolerated the interventions well.

## 3 Result

### 3.1 Cohort Disposition

Figure 1 presents the flow of participants through the trial. A total of 35 individuals were assessed for eligibility, of whom 15 were excluded: 4 did not meet the inclusion criteria and 11 declined to participate. Twenty participants were randomized to the intervention (*n* = 10) or placebo (*n* = 10) arms, and all initiated their assigned treatment. At follow‐up, 9 participants in the probiotic group and 8 in the placebo group remained available for primary outcome assessment. In the probiotic group, one participant missed blood sampling and another was excluded due to insufficient capsule intake, resulting in 8 participants analyzed for immunological outcomes. In the placebo group, two participants missed blood sampling, leaving 6 participants for final analysis. Attrition was therefore mainly attributable to missed blood sampling and adherence issues.

### 3.2 Clinical Characteristics and Inflammatory Profile of Psoriatic Arthritis Patients: Insights From Topographical Data

Table 1 provides a comprehensive overview of the clinical data for patients with psoriatic arthritis, focusing on disease characteristics, skin lesions, inflammatory markers, disease activity, and topography information. This table presents a detailed snapshot of key clinical features among psoriatic arthritis patients aged 32–59 years participating in this study. Several patients exhibited plaque psoriasis in various body regions, illustrating the diverse skin involvement associated with this condition. Additionally, including topographical information enhanced our understanding of skin lesion distribution and severity across the patient population.

**TABLE 1:** Clinical characteristics of psoriatic arthritis patients. This table summarizes the disease characteristics, skin lesion topography, inflammatory markers (CRP levels), and disease activity (DASPA scores) for patients with psoriatic arthritis. It highlights the diversity of skin involvement and provides insights into joint involvement and inflammatory status within the study population. CRP levels exceeding 10 mg/L indicate inflammation, with higher levels associated with more severe disease activity.

Patient ID	Age	Sex	Joint Involvement	DASPA Score	Body area affected	Skin lesion type	CRP (mg/mL)	Disease duration (years)
01	57	F	Wrist, knees, ankle	20	—	—	1	6
02	38	F	Knees	20	Feet	Plaque	1	3
03	55	F	Wrist, knees, hip, fingers	28	Nails, hands	Plaque	2	5
04	43	F	Shoulders, knees, hips, fingers	24	Feet	Plaque	2	2
05	39	F	Wrist, knees, ankle, shoulders	23	—	—	1	3
06	37	M	Wrist, knees, ankle	10	—	—	1	5
07	40	F	Wrist, shoulders	25			2	2
08	59	F	Wrist, shoulders, knees, ankles	23	—	—	1	6
09	44	F	Knees	19	—	—	3	1
10	35	F	Knees, fingers	15	—	—	2	3
11	56	F	Knees, elbow fingers	27			6	1
12	42	F	Knees, wrist hips	24	—	—	2	3
13	55	F	Fingers	13	—	—	1	6
14	32	F	Knees, wrist hips, elbow fingers	22	—	—	0.5	2

#### 3.2.1 Assessment of Baseline Homogeneity in Treatment and Placebo Groups

The average quantitative data and the information in Table 2 demonstrate that our study found no significant differences in age, DASPA scores, or CRP levels between the patient and placebo groups. This suggests that the baseline characteristics of both groups were comparable, reinforcing the validity of the treatment evaluation results. By establishing these measures, we can more confidently attribute any observed effects to the treatment rather than baseline disparities.

**TABLE 2:** Mean ± SD of age, DASPA scores, and CRP levels in patient and placebo groups.

Group	Age (years)	DASPA score	CRP level (mg/L)
Patient group	45 ± 8.7	21.3 ± 5.0	1.5 ± 0.7
Placebo group	44.0 ± 11.1	20.2 ± 5.9	2.3 ± 2.1

*Note:* The table summarizes the average age, DASPA scores, and CRP levels, along with their standard deviations, for both the patient group and the placebo group.

### 3.3 Frequency of Th1, Th2, and B Cells in PsA and Placebo Group

**FIGURE 2:**
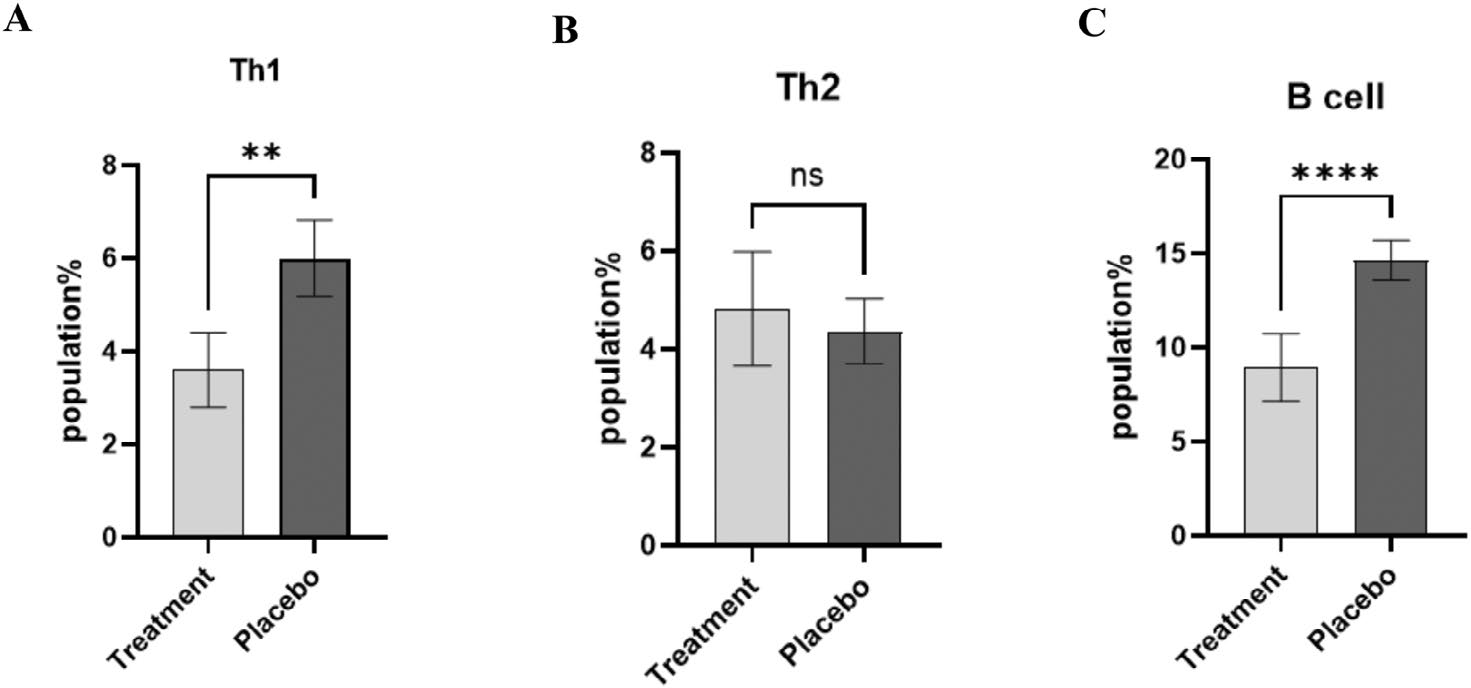
Comparison of immune cell populations in patients with psoriatic arthritis (PsA) and placebo‐treated controls. Results are presented as mean ± standard deviation (SD). (A) Intracellular production of IFN‐γ in CD4+ T cells was significantly lower in PsA patients (3.6% ± 0.8) than in the placebo group (6% ± 0.82; *p* < 0.001). (B) IL‐4 production in Th2 cells showed *no significant* differences between placebo‐treated patients (4.8% ± 1.1) and those receiving probiotics (4.3% ± 0.6; *p* < 0.54). (C) A significant decline in CD19+ B cell populations was observed in PsA patients (8.9% ± 1.7) compared to the placebo (14.6% ± 1.05; *p* < 0.0001). The statistical analysis was performed using the Mann–Whitney U test, and statistical significance compared to the placebo is indicated as follows: ****p < 0.0001, ***p < 0.001, **p < 0.01, and *p < 0.05.

As illustrated in Figure 2, the PsA cohort demonstrated a significant decrease in the percentage of CD4+ T cells that produce IFN‐γ. Only 3.6% ± 0.8 of Th1 cells expressed this cytokine in treated PsA patients with probiotics, compared to 6% ± 0.82 in the placebo group (*p* < 0.001, Figure 2A). In assessing CD4+ T cells producing IL‐4 (Th2), the analysis showed that the proportion of Th2 in the treatment group (4.8% ± 1.1) compared to those who received placebo (4.3% ± 0.6; *p* < 0.54, Figure 2B) was not statistically significant. Among the lymphocyte population, there was also a significant decline in the proportion of CD19+ B cells, with the placebo group exhibiting 14.6% ± 1.05 CD19+ cells compared to 8.9% ± 1.7 in patients with PsA (*p* < 0.0001, Figure 2C).

### 3.4 Serum Levels of Cytokines

#### 3.4.1 IFNγ Levels

**FIGURE 3:**
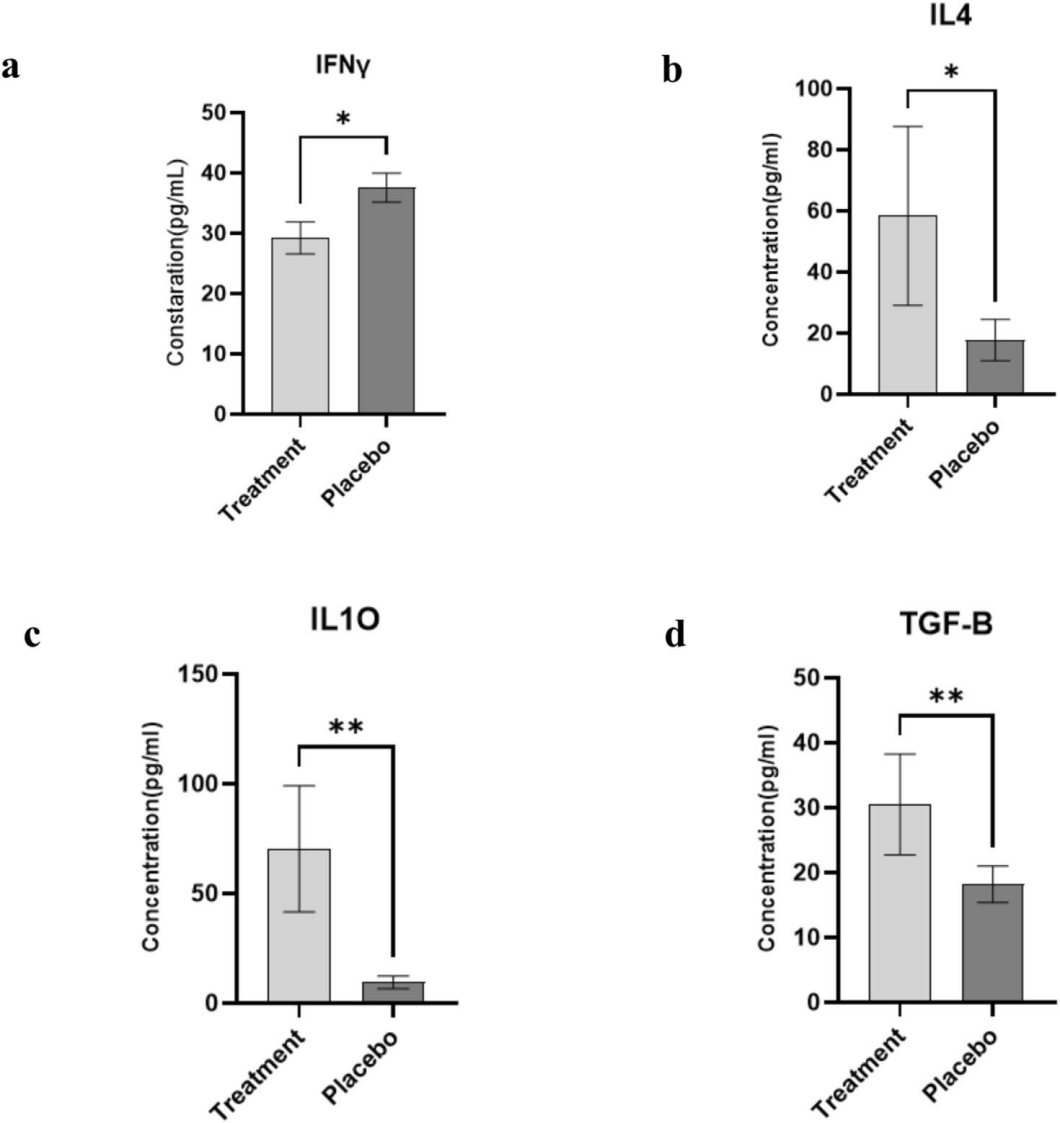
Cytokine concentrations in serum from psoriatic arthritis patients. Results are presented as mean ± standard deviation (SD). This figure illustrates the serum concentrations of IFNγ, IL‐4, IL‐10, and TGF‐β in patients with psoriatic arthritis (PsA) who received probiotic treatment compared to a control group given a placebo. (a) Notably, the treatment group showed a significantly lower average IFNγ concentration of 29.3 ± 2.6 pg/mL (range: 26.1–30.8 pg/mL), while the placebo group had an average of 37.5 ± 2.4 pg/mL (range: 35.7 to 40.3 pg/mL) (*p* = 0.016). (b) Patients in the probiotic group exhibited significantly higher IL‐4 levels, averaging 58.3 ± 29.2 pg/mL, in contrast to the placebo group's mean of 17.6 ± 6.7 pg/mL (*p* = 0.0117). (c) Similarly, serum IL‐10 levels were markedly elevated in the probiotic cohort, where the mean concentration was substantially greater than that of the placebo group, which measured 9.4 ± 2.8 pg/mL (*p* = 0.0032). (d) TGF‐β levels also significantly increased in the probiotic group, reaching a mean of 30.48 ± 7.7 pg/mL, compared to only 18.1 ± 2.7 pg/mL in the placebo group (*p* = 0.0073). The symbols ** and * indicate statistical significance compared to the placebo group, with *p* < 0.01 and < 0.05, respectively.

In assessing the impact of probiotics on patients with psoriatic arthritis over 12 weeks, levels of the cytokine IFNγ were measured in both the treatment and placebo groups. The treatment group demonstrated a significantly lower average IFNγ concentration of 29.3 ± 2.6 pg/mL (with individual values ranging from 26.1 to 30.8 pg/mL), compared to the placebo group, which had an average of 37.5 ± 2.4 pg/mL (values between 35.7 and 40.3 pg/mL). Statistical analysis revealed a significant difference between the two groups, with a *p*‐value of 0.016, indicating a notable reduction in IFNγ levels in the probiotic group (Figure 3a).

#### 3.4.2 IL‐4 Levels

Among the patients receiving probiotics, the mean serum concentration of IL‐4 was measured at (58.3 ± 29.2 pg/mL), with values spanning from 12.7 to 92.6 pg/mL. In contrast, participants in the placebo arm demonstrated a significantly lower mean IL‐4 level of 17.6 ± 6.7 pg/mL. Statistical analysis confirmed that the probiotic group had markedly elevated IL‐4 levels compared to the placebo group, with a *p*‐value of 0.0117 (Figure 3b).

#### 3.4.3 IL‐10 Levels

The serum level of IL‐10 in the probiotic cohort averaged (70.3 ± 9.4 pg/mL), encompassing a range from 28.1 to 99.89 pg/mL. This was significantly greater than the mean concentration in the placebo group, which was 9.4 ± 2.8 pg/mL (*p* = 0.0032) (Figure 3c). These results indicate that probiotic supplementation may significantly boost IL‐10 production in patients with psoriatic arthritis.

#### 3.4.4 TGF‐β Levels

In the probiotic group, the serum level of TGF‐β was found to be (30.48 ± 7.7 pg/mL), with measurements ranging from 18.8 to 40.6 pg/mL. Conversely, the placebo group exhibited a mean TGF‐β level of (18.1 ± 2.7 pg/mL). A statistically significant difference was identified between the two groups, with TGF‐β levels being considerably higher in the probiotic participants (*p* = 0.0073) (Figure 3d).

### 3.5 Overview of Immune Cell Ratios in Probiotic and Placebo Treatments

The monocyte (Mon/PBMC) proportions were similar between the probiotic treatment and placebo groups, with no significant differences observed. Additionally, while lymphocyte proportions (Lym/PBMC), the lymphocyte‐to‐monocyte ratio (Lym/Mon), and the Th1 to Th2 ratio were slightly lower in the treatment group than in the placebo group, these differences were insignificant. Conversely, the T helper to B cell ratio (Th/B) was slightly higher in the probiotic group than in the placebo group, but again, this difference was not statistically significant. These findings are reported only for descriptive purposes (Table 3).

**TABLE 3:** Immune cell populations and proportions in PBMC from psoriatic arthritis patients receiving probiotics versus placebo. Bar graphs represent the mean ± standard deviation (SD) for the proportion of each immune subset, including Mon/PBMC, Lym/PBMC, Lym/Mon, Th1/Th2, and Th/B cells, in both treatment and placebo arms. No statistically significant differences were observed between corresponding treatment and placebo groups (*p* > 0.05, one‐way ANOVA followed by post hoc multiple comparisons and unpaired t‐tests where needed).

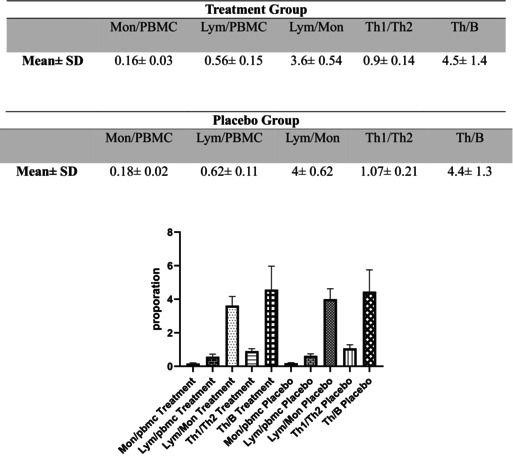

### 3.6 Immunoprofile Changes Induced by Multi‐Strain Probiotic Capsule

In this study, we compared cytokine and immune‐cell outcomes between the probiotic and placebo groups. The probiotic group showed statistically significant improvements across several immunological markers. Overall, several cytokine and immune‐cell parameters favored probiotic supplementation, with notably larger differences observed for IL‐10 and TGF‐β, while CD4^+^ IFN‐γ T cells and IFN‐γ levels also demonstrated statistically meaningful reductions in the probiotic group compared with placebo (Table 4).

**TABLE 4:** Cytokine and immune‐cell outcomes by treatment group (*N* = number of participants per group). For each outcome, the effect size corresponds to the difference (probiotic − placebo). Negative values indicate lower levels in the probiotic group; positive values indicate higher levels in the probiotic group.

Outcome	Group	Included (*N*)	Available data (*N*)	Mean ± SD	Effect size (probiotic − placebo)	95% CI	*p*
CD4^+^ IFN‐γ T cells (%)	Probiotic	8	6	3.6% ± 0.8	−2.397	−14.11 to −2.53	0.001
	Placebo	6	6	6% ± 0.82			
B cells (%)	Probiotic	8	8	8.9% ± 1.7	−5.699	−7.50 to −3.89	0.0001
	Placebo	6	6	14.6% ± 1.0			
Th2 cells (%)	Probiotic	8	8	4.3% ± 0.6	0.453	−1.186 to 2.09	0.54
	Placebo	6	6	4.8% ± 1.1			
IFN‐γ (pg/ml)	Probiotic	8	8	29.3 ± 2.6	−8.32	−14.11 to −2.53	0.016
	Placebo	6	6	37.5 ± 2.4			
IL‐10 (pg/ml)	Probiotic	8	8	70.3 ± 28.6	60.90	27.04 to 94.77	0.0032
	Placebo	6	6	9.4 ± 2.8			
TGF‐β (pg/ml)	Probiotic	8	8	30.48 ± 7.7	12.30	4.12 to 20.48	0.0073
	Placebo	6	6	18.1 ± 2.7			
IL‐4 (pg/ml)	Probiotic	8	8	58.3 ± 29.2	40.70	11.00 to 70.39	0.0117
	Placebo	6	6	17.6 ± 6.7			

## 4 Discussion

In recent years, numerous studies have established a connection between probiotics and psoriasis. However, this clinical trial is the first to examine the impact of a probiotic mixture administered to patients with psoriatic arthritis, assessing various immune cell populations and cytokines. To ensure the validity of our findings, we controlled for confounding factors, including antibiotic use, disease severity, and the presence of other allergic conditions.

In this clinical trial, we enrolled patients with a confirmed diagnosis of psoriatic arthritis as determined by a rheumatologist. All participants exhibited joint inflammation and other symptoms characteristic of psoriatic arthritis. Additionally, a subset of patients presented with plaque psoriasis (Table 1). The inclusion of individuals with both psoriatic arthritis and plaque psoriasis allowed us to explore and comprehensively investigate the effects of probiotic supplementation on joint and skin inflammation in patients with psoriatic arthritis and plaque psoriasis and various aspects of these interconnected inflammatory conditions. To ensure the reliability and validity of our study, we selected participants with a DAPSA score below 28, indicating low to moderate disease activity. This decision was made to avoid enrolling individuals who typically require systemic therapy and present with more severe disease complications. As shown (Table 2), there were no significant differences in age, DAPSA scores, or CRP levels between the two groups at baseline. The comparable age distribution allowed us to attribute observed changes during the study to the treatment rather than age‐related factors, which could influence disease progression and response to treatment. Similarly, the lack of significant differences in baseline DAPSA scores and CRP levels indicated that both groups shared similar levels of disease activity and inflammation before the intervention.

The results of our study reveal a significant reduction in the Th1 cell population (Figure 2A, *p* < 0.001) and IFN‐γ (Figure 3d, *p* < 0.01) within the treatment group receiving the probiotic blend, in comparison to the placebo group. This finding indicates that the probiotic blend, when used as an adjunct therapy alongside standard treatments (Methotrexate, prednisolone, and sulfasalazine), may contribute to a decrease in the pro‐inflammatory Th1 cells, which play a key role in the development of psoriatic arthritis. These findings align with a study by Jeong et al., where oral administration of the probiotic strain *Lactobacillus pentosus* significantly reduced skin lesions in an imiquimod‐induced psoriasis‐like mouse model. In addition to decreased expression of pro‐inflammatory cytokines, *L. pentosus* treatment also reduced spleen weight and the number of CD4+ T cells in the spleen of imiquimod‐treated mice (Chen et al. 2017). Further supporting evidence comes from Fan et al., where the administration of specific probiotic strains in a collagen‐induced arthritis model resulted in reduced levels of IFN‐γ and TNF‐α in synovial fluid serum and an increased proportion of Treg cells. This suggests that probiotics could potentially mitigate Th1‐mediated inflammation associated with autoimmune arthritis (Fan et al. 2021).

Our study demonstrates a reduction in the B‐cell population following treatment with a probiotic mixture (Figure 2B, *p* < 0.0001). This finding and the increased expression of the anti‐inflammatory cytokine IL‐10 (Figure 3b, *p* = 0.0032) suggest that the probiotic mixture may exert its immunomodulatory effects through modulating B‐cell response.

B cells play a critical role in the adaptive immune response, particularly in antibody production and immune memory generation (Ahmadishoar et al. 2025; Karimi et al. 2024). The decrease in B‐cell population observed in our study could indicate that the probiotic mixture modulates B‐cell activation, differentiation, or proliferation. In light of Hayashi et al.'s study, which reported a decrease in IL‐10 regulatory B cells in psoriasis patients (Hayashi et al. 2016), our findings on the effects of the probiotic mixture become even more compelling. In our psoriatic arthritis patient study, the increase in IL‐10 production caused by the probiotic mixture could be responsible for decreased B‐cell numbers, given IL‐10's role in suppressing B‐cell activation, proliferation, and differentiation. Animal studies further support the essential role of IL‐10‐producing B cells in modulating immune and inflammatory responses, as mice lacking these regulatory B cells develop severe arthritis (Mavropoulos et al. 2017). Additionally, there was an increase in TGF‐β levels following probiotic administration in psoriasis patients compared to the placebo group (Figure 3b, *p* = 0.0072). This suggests that the probiotic mixture may modulate immune and inflammatory responses, at least in part, by enhancing TGF‐β production. It stands to reason that the observed decrease in the B‐cell population aligns with TGF‐β's inhibitory effects on B cells. In a related study by Esmaili et al., probiotics were found to modulate TGF‐β production and alleviate the severity of skin inflammation in psoriasis. They demonstrated the potential of probiotics in managing inflammatory skin conditions by modulating the immune response and altering the gut microbiome. A key finding of their study was the ability of probiotics to increase TGF‐β production, which can promote the conversion of naïve CD4 + non‐Tregs into Tregs (Atabati et al. 2020).

Our results showed a non‐significant increase in the Th2 cell population in the probiotic‐treated group compared to the placebo group after 12 weeks (Figure 1B, *p* = 0.54). While this change was not statistically significant, it may suggest a potential immunomodulatory trend that warrants further investigation, and it may reflect a small sample size or short duration. Nonetheless, previous studies have demonstrated that specific probiotic strains, such as *Lactobacillus casei* , may influence the Th1/Th2 balance. For instance, Vaghef's study reported that probiotic supplementation in rheumatoid arthritis (RA) patients led to a Th2‐dominant immune shift, accompanied by improvements in disease activity. This highlights the potential of probiotics to modulate immune responses through cytokine regulation (Vaghef‐Mehrabani et al. 2018). Cytokine IL‐4, a crucial player in the Th2 immune response, has been closely linked to the immunomodulatory effects of probiotics. Prens et al. demonstrated that IL‐4 downregulates the expression of antimicrobial protein hBD2 and induces phospho‐STAT6 in psoriatic skin, contributing to reduced inflammation and a more balanced immune response. Furthermore, IL‐4 upregulates the expression of the transcription factor GATA3 in epidermal cells and keratinocytes, promoting Th2 immune responses while counteracting the proinflammatory Th1 and Th17 responses commonly observed in psoriasis (Onderdijk et al. 2015). Notably, we observed a significant increase in IL‐4 levels in the probiotic‐treated group (Figure 3a, *p* = 0.0117). This is a particularly important finding as IL‐4 plays a vital role in modulating immune responses and fostering an anti‐inflammatory environment. The substantial elevation in IL‐4 levels in the treatment group indicates that probiotic supplementation may influence immune responses by enhancing the production of anti‐inflammatory cytokines, which could help alleviate inflammation and promote a more balanced immune system.

Emerging evidence suggests that probiotics may play a complementary role in the management of PsA, potentially supporting gut health and modulating immune responses when used alongside standard therapies, rather than replacing them (Bonomo et al. 2025; Grinnell et al. 2020). Through their impact on gut microbiota composition and related nutritional pathways, such as short‐chain fatty acid production and intestinal barrier integrity, probiotics may support systemic immune balance in psoriatic conditions (Fallahi et al. 2025). However, larger, well‐powered studies are needed to validate these mechanisms and establish clinical relevance. Furthermore, our findings must be interpreted with caution due to the small sample size of this pilot study. With only 14 participants completing the trial, the statistical power was limited, increasing the risk of error and reducing the generalizability of the results. While observed trends, including the non‐significant increase in Th2 cells, may indicate biologically relevant effects, larger, well‐powered studies are needed to validate these findings, clarify underlying mechanisms, and determine clinical relevance.

## 5 Conclusion

Our study showed that administering a mixture of probiotic tablets over 12 weeks significantly reduced the populations of Th1 and B cells in the treatment group of psoriasis arthritis patients. Further analysis revealed a decrease in the inflammatory cytokine IFNγ and an increase in the anti‐inflammatory cytokines IL‐10, TGF‐β, and IL‐4. These results indicate that the probiotic formulation possesses anti‐inflammatory properties, suggesting potential adjunct therapy for managing immune‐mediated diseases. Given the side effects and potential treatment resistance associated with current anti‐inflammatory drugs, probiotics could serve as a complementary or alternative treatment strategy. Their use alongside targeted therapies or as a preventive measure may be advantageous for patients with these conditions.

### 5.1 Study Limitations

This is the first clinical trial to evaluate the effectiveness of a probiotic mixture on the immune cell population and cytokine levels in patients with psoriatic arthritis, and no prior data exist on this specific probiotic cocktail. The small sample size limits the generalizability of the findings and precludes meaningful subgroup analyses, such as stratification by psoriasis severity. Additional limitations include the use of a single probiotic dose, a relatively short 12‐week intervention period, recruitment from a single clinical center, and the absence of dietary control during the study. Since diet can significantly influence gut microbiota composition, future trials should incorporate dietary monitoring or control to better isolate the effects of probiotic supplementation. Further studies should also enroll larger and more diverse populations and examine dose escalation, longer treatment durations, and comparisons across different probiotic strains to build on these pilot findings.

### 5.2 Adverse Events

In this assessment, there were no side effects associated with the interventional medication, and all patients completed the 12‐week follow‐up. No relevant unintended effects were reported with either the probiotic or placebo intake. All reported or observed adverse effects were assessed and documented by the study physician. In this assessment, no side effects were associated with the interventional medication, and all patients completed the follow‐up period within 3 months. Additionally, no severe side effects occurred in either study group during the intervention, and no patients were withdrawn due to treatment‐related side effects.

## Author Contributions

**Ahmed Hussein Hasan Alshihmani:** methodology (equal), project administration (equal), writing – original draft (equal). **Hanieh Kolahdooz:** project administration (equal), writing – original draft (equal). **Mahmoud Mahmoudi:** supervision (equal). **Zahra Rezaieyazdi:** project administration (equal). **Ramiar Kamal Kheder:** editing – original draft (equal). **Nafiseh sadat Tabasi:** project administration (equal). **Afsane Fadaee:** project administration (equal). **Seyed‐Alireza Esmaeili:** methodology (equal), project administration (equal), writing – original draft (equal), supervision (equal), Editing – original draft (equal).

## Conflicts of Interest

The authors declare no conflicts of interest.

## Supporting information


**Data S1:** fsn371132‐sup‐0001‐DataS1.pdf.

## Data Availability

The data will be made available on request.
