# Supplementary material for: Immunomodulatory Effects of Multi‐Strain Probiotic Capsules for Psoriatic Arthritis: A Pilot Double‐Blind Randomized Controlled Trial
Source: Food Sci Nutr. 2025 Nov 5;13(11):e71132. doi: 10.1002/fsn3.71132 (PMC12588955; doi:10.1002/fsn3.71132)
Supplement: Supplementary file 1 — Data S1: fsn371132‐sup‐0001‐DataS1.pdf. [file FSN3-13-e71132-s001.pdf]

ZIST TAKHMIR PHARMACEUTICAL Co	ZIST TAKHMIR PHARMACEUTICAL COMPANY<br>Certificate of Analysis (Finished Product)	Form No: ZT.QC.GEN.F.013<br>Revision No: 01

**Product Name:** LactoCare® Capsule			
**Batch No:**LC0402	**Mfg Date:** 07-2025	**Rev No of Spc:**	02
**Lab No:**04-0189	**Exp. Date:** 07-2027	**Page:**	1 of 1
**Batch Size:** 34000 Boxes	**Date of Report:** 07-22-2025		
**Storage Condition:** Preserve in well-closed Containers,Store in 2-8 C°			

Test	Acceptance Criteria	Result	Reference
**Description**	Yellowish Fine Powder, Filled in Gelatin Capsule with white body-white cap, Size 0	Conform	In-House
**Loss on Drying**	NMT 5.0%	4.0%	In-House
**Disintegration Time**	NMT 30 min	4 min	In-House
**Average Weight (mg)**	450 mg ±7.5% (416-484 mg)	452.6 mg	In-House
**Net Weight (mg)**	350 mg ±7.5% (324-376 mg)	352.6 mg	In-House
**Weight Variation of Dietary Supplements**	**A:** No Capsules differ from the average weight of 20 Capsules by more than 10%	Conform	USP 46/NF41
	**B:** If case A is reject; the net weights of NMT 2 of the Capsules differ from the average net weight of 20 capsules by more than 10% and no case does the difference exceed 25%	Conform	
**Assay (Total Probiotic bacteria count)**	MT 10^8 CFU/g	1.6×10^9 CFU/g	ISIRI (4721)<br>ISIR (19459)
**Non lactic acid bacteria count**	NMT 10^3 CFU/g	None	ISIRI (8248)
**Total Yeast & Mold Count**	LT 100 CFU/g	None	ISIRI (10154)<br>ISIR (19459)
**Total Coliforms**	NMT 3 MPN/g	None	FDA BAM<br>(Chapter 4)
**Enterobacteriaceae**	LT 10 CFU/g	None	ISIRI (2461-1,2)<br>ISIR (19459)
Escherichia Coli	Absent	Conform	ISIRI (5234)<br>ISIR (19459)
Bacillus cereus	LT 100 CFU/g	None	ISIRI (2324)<br>ISIR (19459)
Staphylococcus aureus	Absent	Conform	ISIRI (6806-1-2-3)<br>ISIR (19459)
Salmonella Species (25g)	Absent	Conform	ISIRI (4413)<br>ISIR (19459)
Listeria monocytogenes	Absent	Conform	ISIRI (8035-1)<br>ISIR (19459)
Clostridium Species	LT 10	None	ISIRI (9432)<br>ISIR (19459)
Pseudomonas aeruginosa	Absent	Conform	USP46/NF41
**Final Result**	**According To: In-House, Monograph**	☑ **Approved**	☐ **Rejected**

Comment:

Name/Date/Signature of physicochemical QC Head:<br>Approved<br>۱۴۰۴/۰۴/۳۱<br>(Physicochemical test)<br>Quality Control<br>7.22.2025	Name/Date/Signature of Microbial QC Head:<br>Approved<br>۱۴۰۴/۰۴/۳۱<br>(Microbial test)<br>Quality Control<br>7.22.2025
Name/Date/Signature of QC Manager:<br>PASSED<br>QC Lab<br>07.23.2025	Name/Date/Signature of Authorized Person:<br>ZIST TAKHMIR<br>APPROVED<br>۰۵.۰۵.۰۴
